# Functions of PDE3 Isoforms in Cardiac Muscle

**DOI:** 10.3390/jcdd5010010

**Published:** 2018-02-06

**Authors:** Matthew Movsesian, Faiyaz Ahmad, Emilio Hirsch

**Affiliations:** 1 Department of Internal Medicine/Division of Cardiovascular Medicine, University of Utah, Salt Lake City, UT 841132, USA; 2 Vascular Biology and Hypertension Branch, Division of Cardiovascular Sciences, National Heart, Lung and Blood Institute, Bethesda, MD 20892, USA; ahmadf@nhlbi.nih.gov; 3 Department of Molecular Biotechnology and Health Sciences, Center for Molecular Biotechnology, University of Turin, 10126 Turin, Italy; emilio.hirsch@unito.it

**Keywords:** cyclic nucleotides, cAMP, cGMP, phosphodiesterase, PDE3, intracellular signalling, heart

## Abstract

Isoforms in the PDE3 family of cyclic nucleotide phosphodiesterases have important roles in cyclic nucleotide-mediated signalling in cardiac myocytes. These enzymes are targeted by inhibitors used to increase contractility in patients with heart failure, with a combination of beneficial and adverse effects on clinical outcomes. This review covers relevant aspects of the molecular biology of the isoforms that have been identified in cardiac myocytes; the roles of these enzymes in modulating cAMP-mediated signalling and the processes mediated thereby; and the potential for targeting these enzymes to improve the profile of clinical responses.

## 1. Introduction

Cyclic nucleotide phosphodiesterases regulate intracellular signalling by hydrolysing cAMP and/or cGMP. Enzymes in the PDE3 family of phosphodiesterases are dual-specificity enzymes with high affinities for both cAMP and cGMP but much higher turnover rates for cAMP [1,2,3]. In cardiac muscle, these enzymes have been studied principally in the context of their role in regulating cAMP-mediated signalling, and this is the focus of our review.

Several isoforms of PDE3 are expressed in cardiac myocytes, and PDE3 inhibitors are used therapeutically to potentiate cAMP-mediated signalling in patients with heart failure. In the short term, these agents have the desired action of increasing myocardial contractility, but their long-term administration has been shown in several clinical trials to increase cardiovascular mortality [4,5,6,7,8,9,10]. This frustrating combination of beneficial and adverse effects of PDE3 inhibition presents a challenge that remains to be solved. Here we review the function of PDE3 isoforms in cardiac muscle and raise possibilities for targeting these isoforms so as to achieve more satisfying clinical outcomes. 

## 2. The PDE3 Family of Cyclic Nucleotide Phosphodiesterases

Cyclic nucleotide phosphodiesterases comprise a superfamily of enzymes. As of now, more than 50 mammalian isoforms have been described and classified into eleven gene families (PDE1 through PDE11) defined on the basis of sensitivity to pharmacologic inhibitors, kinetic activity, and regulatory mechanisms [11]. PDE1, 2, 3, 10, and 11 hydrolyse both cAMP and cGMP; PDE4, PDE7, and PDE8 selectively hydrolyse cAMP; and PDE5, PDE6, and PDE9 selectively hydrolyse cGMP [11,12,13]. The N-terminal regulatory regions of phosphodiesterases contain sequences involved in post-translational modifications and protein–protein interactions that target the enzymes to specific functional compartments. Transcription start sites and alternative splicing lead to the generation of multiple different isoforms of the same family.

**Figure 1:**
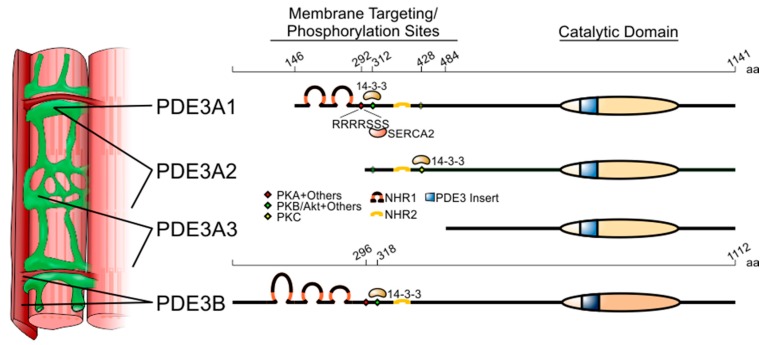
Structure and subcellular localisation of the PDE3 genes and their variants. Length in amino acids (aa) is provided at the top of the two PDE3 isoforms. PDE3A1 is translated from the second AUG codon of the open-reading frame found in the PDE3 mRNA. While the longest variant of PDE3A, PDE3A1, is mainly localised to the sarcoplasmic reticulum, PDE3A2 and PDE3A3 are found both in membranes and cytoplasm. PDE3B is mainly localised to plasma membrane invaginations known as T tubules. Coloured diamonds indicate phosphorylation sites. Selected PDE3-interacting proteins are listed where the precise binding sequences are known. Membrane-associated N-terminal hydrophobic regions 1 and 2 (NHR1 and 2) are depicted as loops. The catalytic domain, highly conserved between PDE3A and PDE3B, is indicated as a striped oval that includes the 44-amino-acid insert characteristic of PDE3 isoforms.

Enzymes in the PDE3 family are transcribed from two genes, PDE3A and PDE3B [14,15]. In the case of PDE3A, three isoforms (some prefer the term ‘variants’) are generated by transcription from alternative starts sites in the gene, yielding two mRNAs, as well as translation from alternative start sites in the smaller mRNA (Figure 1) [16]. As a result of these N-terminal ‘deletions’, the amino-acid sequences of these three isoforms differ only with respect to the lengths of their N-terminal sequences. PDE3A1 (length: 996 amino acids; MW: 109,980), which is transcribed from an upstream start site and translated from the second AUG in the PDE3A open reading frame—a possibly misleading term in this case, as no isoform translated from the first AUG has been described—has an N-terminal sequence containing hydrophobic loops that insert into intracellular membranes [17,18], as well as three sites of phosphorylation that regulate protein–protein interactions [19,20,21]. PDE3A2 (length: 842 amino acids; MW: 93,600) is transcribed from a downstream site in exon 1 and translated from the fourth AUG in the PDE3A open reading frame; it lacks the most N-terminal phosphorylation site and the transmembrane hydrophobic loops of PDE3A1. PDE3A3 (length: 659 amino acids; MW: 73,720) is translated from the same mRNA as PDE3A2 and lacks all of the hydrophobic loops and the upstream phosphorylation sites. These three isoforms are essentially indistinguishable with respect to their basal catalytic activity and their sensitivity to catalytic site inhibitors [22]. At this time, only one isoform of PDE3B (length: 1112 amino acids; MW: 124,333) has been described [15]. Like PDE3A1, its N-terminal sequence contains hydrophobic loops (six for PDE3B, as compared to four for PDE3A1) and phosphorylation sites, and its C-terminal sequence contains its catalytic region [23,24]. The sequence of the catalytic region of PDE3B is >80% identical to that of PDE3A (both contain a 44-amino-acid insert absent from other phosphodiesterase families), and its catalytic activity and inhibitor sensitivity are similar to those of PDE3A; the remainder of the PDE3B sequence is 20–30% identical to that of PDE3A [3]. 

## 3. Intracellular Localisation of PDE3A and PDE3B in Cardiac Myocytes and Their Protein–Protein Interactions

Cyclic nucleotide-mediated signalling is highly compartmentalised in cardiac myocytes, and the roles of individual phosphodiesterases in regulating cAMP- and cGMP-mediated signalling depend upon their intracellular localisation. While PDE3A and PDE3B are both expressed in cardiac myocytes (PDE3A more abundantly [25]), their intracellular distributions are distinct, with PDE3A localised mainly to the sarcoplasmic reticulum and PDE3B to T tubules in proximity to mitochondria [26] (Figure 1). PDE3 activity is also associated with nuclear membranes in cardiac myocytes [27], though the specific isoforms have not been delineated. Furthermore, in subcellular preparations from cardiac muscle, PDE3A1 is recovered solely in microsomal fractions, while PDE3A2 and PDE3A3 are recovered in cytosolic as well as microsomal fractions [16]. This corresponds to studies in cells transfected with PDE3A1- and PDE3B-derived constructs that show that the N-terminal hydrophobic loops in these isoforms direct the insertion of these proteins into lipid membranes [17,18].

### 3.1. PDE3A

The precise intracellular localisation of PDE3 isoforms depends upon their interactions with anchoring, scaffold, and adaptor proteins that recruit the enzyme to multiprotein signalling complexes [28,29,30,31,32,33]. Localised A-kinase anchoring proteins (AKAPs) tether protein kinase A (PKA) and other signalling proteins—adenylyl cyclases, phosphatases, Epacs, PDEs and other effector molecules—to ‘signalosomes’ that allow selective phosphorylation of individual PKA substrates [31,32,33,34]. 

**Figure 2:**
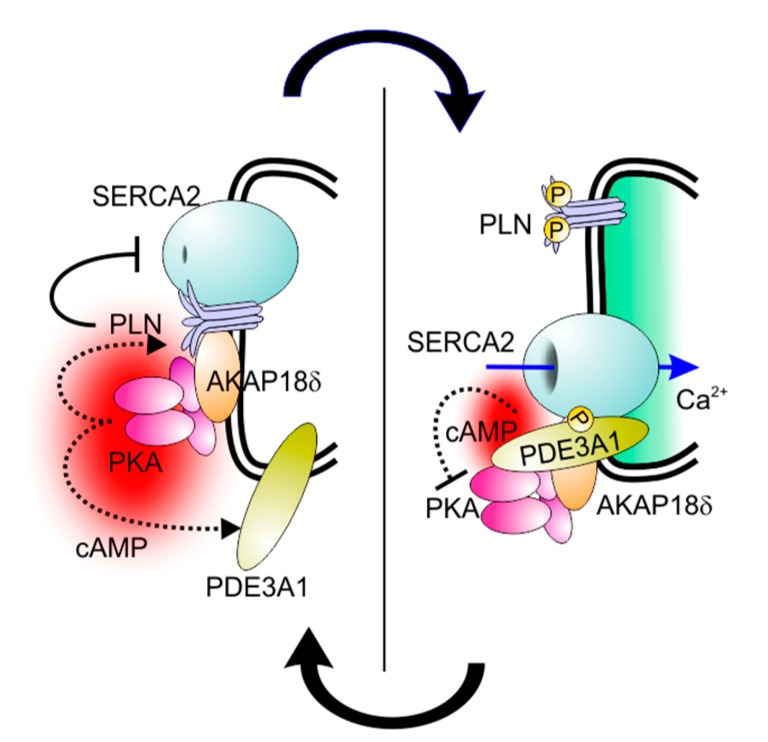
Control of SERCA2 activity by PDE3A. Left panel: Under resting conditions, phospholamban (PLN) binds to SERCA2, whose activity it inhibits, as well as AKAP18δ on the cytoplasmic surface of the sarcoplasmic reticulum (double black line). Upon β-adrenergic receptor-stimulated cAMP production, PKA associated with AKAP18δ is activated and phosphorylates PLN (upper dotted line) and PDE3A1 (lower dotted line). This leads to the condition shown in the right panel, where phosphorylated PLN detaches from SERCA2 and loses its inhibitory action. As a consequence, SERCA2-dependent Ca^2+^ uptake into the lumen of the sarcoplasmic reticulum is stimulated (encircled by the double black line). At the same time, phosphorylated PDE3A1 binds to SERCA2 and increases its cAMP-hydrolytic activity, which limits the extent to which β-adrenergic receptor-mediated signalling amplifies intracellular Ca^2+^ cycling.

PDE3 has long been known to be associated with the sarcoplasmic reticulum of cardiac myocytes [35,36]. Confocal microscopy studies more recently demonstrated co-localisation of PDE3A with SERCA2, AKAP18, phospholamban, and desmin in the Z-bands of cardiac myocytes [37,38]. PDE3A was found to be a constituent of a multiprotein complex in the sarcoplasmic reticulum containing AKAP18, phospholamban, and SERCA2 [38,39]. Addition of cAMP to microsomes from human heart results in the phosphorylation of phospholamban by endogenous PKA; this leads to a dissociation of phospholamban from SERCA2 and an increase in SERCA2 activity, and this effect is potentiated by PDE3 inhibition [38] (Figure 2). Although PDE3 and PDE4 have both been found to co-immunoprecipitate with AKAP-based signalosomes from human and mouse myocardium and modulate effects of cAMP on *L*-type Ca^2+^ channels, ryanodine-sensitive Ca^2+^ channels, and SERCA2 [40,41,42,43], only PDE3 inhibition potentiates the PKA-mediated phosphorylation of phospholamban and the consequent stimulation of SERCA2 activity [37]. The role of PDE3A in modulating these effects is the likely explanation for the inotropic actions of PDE3 inhibition. 

Furthermore, while PDE3 isoforms regulate the phosphorylation of other proteins through the cAMP/PKA pathway, they are themselves substrates for protein kinases that modulate their catalytic activity and protein–protein interactions [19,21,44,45,46,47]. The incorporation of PDE3A into the SERCA2 complex is an example. In co-immunoprecipitation experiments, phosphorylation of endogenous PDE3A by PKA increases its interactions with SERCA2, caveolin-3, PKA regulatory subunit (PKARII), PP2A, and AKAP18 [38]. Studies with recombinant proteins showed that phosphorylation of PDE3A by PKA increased its co-immunoprecipitation with SERCA2a and AKAP18, suggesting that PDE3A interacts directly with both proteins in a phosphorylation-dependent manner [38]. Deletion of the N-terminal region of PDE3A1/PDE3A2 blocked PKA-induced phosphorylation of PDE3A and its interaction with recombinant SERCA2. Of particular interest is the sequence RRRRSSS (amino acids 288–294 of the open reading frame, which are found only in PDE3A1), which provides three serines that can be phosphorylated in vitro by different kinases under different conditions [19]. The introduction of serine-to-alanine substitutions at S292-4 identified this sequence as the principal site responsible for regulating the interactions of PDE3A1 with SERCA2 (Figure 1). 

The absence of the S292-4 site from PDE3A2 and PDE3A3 establishes a PDE3A1-specific mechanism for recruitment of PDE3A to the SERCA2 complex. The protein–protein interactions of PDE3 isoforms are distinct in other ways as well, as has been described for the phosphorylation-dependent interactions of PDE3A1, PDE3A2, and PDE3B with 14-3-3 [20,21,48,49,50]. The common sequence of PDE3A1 and PDE3A2 includes two 14-3-3-binding sites: S428, a PKC site; and S312, a PKA/PKB (alternatively referred to Akt) site that resembles S318, a 14-3-3-binding site in PDE3B [48,49] (Figure 1). In vitro, PDE3A1 is preferentially phosphorylated by PKA at S312, whereas PDE3A2 is preferentially phosphorylated by PKC at S428; in preparations from human hearts, PDE3A1 is phosphorylated primarily at S312, while PDE3A2 is phosphorylated primarily at S428 [50]. Furthermore, in transfected HEK293 cells, the phosphorylation-dependent interactomes of PDE3A1 and PDE3A2 are distinct [50]: PDE3A1 interacts with the 5-HT_4(b)_ receptor, for example, while PDE3A2, PDE3A3, and PDE3B do not [51]. These unique protein–protein interactions may provide opportunities for isoform-specific targeting of the protein–protein interactions of individual isoforms.

**Figure 3:**
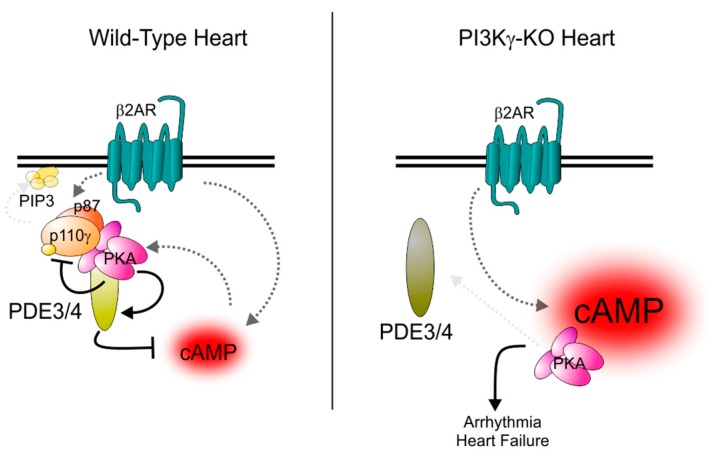
Regulation of PDE3 and PDE4 by the AKAP function of PI3Kγ. Left panel: Regulation of PI3Kγ and phosphodiesterase activity in healthy conditions. Upon stimulation of β-adrenergic receptors, cAMP production is promoted but simultaneously constrained by the activation of a negative feedback loop involving the scaffold function of PI3Kγ that directly binds the PKA holoenzyme containing the RII regulatory subunit as well as PDE3s and PDE4s. PKA activation leads to the phosphorylation of these phosphodiesterases and the consequent increase in their activity that, in turn, reduces cAMP levels. At the same time, PKA phosphorylates and inhibits PI3Kγ, thus blocking the classical PI3K pathway signalling. Right panel: In the absence of PI3Kγ, PKA is displaced from PDE3 and PDE4 enzymes and is unable to efficiently stimulate their activity. As a consequence, cAMP levels rise and cAMP diffuses to compartments that β-adrenergic receptor-mediated signalling does not usually affect.

In cardiac myocytes, PDE3A is also part of a multiprotein complex containing the unconventional AKAP PI3Kγ [52]. While PI3Kγ directly binds the RII subunit of the PKA holoenzyme, how PDE3A contacts the complex is still unclear, though a likely intermediate is the PI3Kγ interactor p84/p87 [53]. Within this complex, PKA exerts a negative feedback regulation by phosphorylating and activating the different associated PDE isoforms, including PDE3A [39]. In line with the role of PDE3A (and PDE4A/B) in controlling SERCA2, loss of the scaffold function of PI3Kγ leads to cAMP elevation and PKA-mediated hyperphosphorylation of phospholamban and *L*-type Ca^2+^ channels [39,54] (Figure 3, left panel). In hearts lacking PI3Kγ and subjected to pressure overload, this effect causes cAMP elevation, contractile dysfunction, and increased mortality due to lethal arrhythmic events such as sustained ventricular tachycardia [39] (Figure 3, right panel). Interestingly, this abnormally increased cAMP accumulation appears to be selectively associated with signalling through β-adrenergic receptors localised to T tubules [55]. This would suggest that the PI3Kγ PKA/PDE complex is part of an ‘insulating’ system that blocks ‘leakage’ of cAMP from this compartment to the sarcoplasmic reticulum [39]. In heart failure, the expression of various elements of the PI3Kγ PKA/PDE3A-containing complex change their stoichiometric ratios, with reduced expression of p84/87 and coincident increased expression of PI3Kγ. This causes a dysfunctional cAMP regulation potentially contributing to the increase in ventricular arrhythmias associated with heart failure [52].

### 3.2. PDE3B

In contrast to PDE3A, PDE3B is mainly located in caveolin-3-rich areas of the plasma membrane and, in cardiac myocytes, in the T tubules (Figure 1). This indicates that the two PDE3 genes are specifically involved in the regulation of spatially and functionally distinct pools of cAMP. In line with this view, while PDE3A is involved in the regulation of contractility, PDE3B appears more connected to the regulation of metabolism. PDE3B is also known to have a role in the liver, pancreatic β cells, and in the brown and white adipose tissue where it is involved in the control of the anti-lipolytic effect of insulin, generally in contrast to β-adrenergic signalling [56,57]. In the heart, the specific inactivation of PDE3A causes alterations in basal cardiac contractility, while genetic ablation of PDE3B has no major impact on contractility but protects the myocardium from ischemic damage [26,37]. Interestingly, the protective effect of PDE3 inhibitors in ischemic heart preconditioning had been known for several years, but the precise nature of the PDE3 isoform only emerged with studies in knockout mice that excluded a role of PDE3A. The specific localisation of PDE3B in T tubules, and particularly both in dyads and in close proximity to mitochondria, suggests that this particular phosphodiesterase plays a role in modulating energy metabolism. Elevation in cAMP concentration in this location might mediate cardioprotective signals that improve mitochondrial function and energy supply during ischaemia/reperfusion, where mitochondrial Ca^2+^ overload and consequent mitochondrial permeability transition (MPT) pore opening, oxidative stress, and apoptosis contribute to injury [58]. In hearts lacking PDE3B, mitochondria are enriched in Bcl-2, produce lower amounts of reactive oxygen species, and show more numerous contacts with T tubules. In response to ischaemia, mitochondria from PDE3B-deficient cardiac myocytes are more resistant to Ca^2+^-induced opening of the MPT pore, and associate with caveolin-3-enriched membrane subfractions containing cardioprotective proteins. The recruitment of these cardioprotective proteins to these subfractions is PKA-dependent, and can be reproduced in wild-type mice by PDE3 inhibition [26]. 

The nature of the localization signal that keeps PDE3B in such spatially and functionally distinct cellular subdomains remains unknown. Various reports indicate that PDE3B can be recruited to different multiprotein complexes with distinct properties. In adipocytes, for example, treatment with insulin increases the phosphorylation of PDE3B associated with internal membranes, promoting its interactions with IRS-1 (insulin receptor substrate-1), IRS-2, PI3K p85 (p85-subunit of phosphoinositide 3-kinase), PKB (protein kinase B), HSP-90 (heat-shock protein 90), 14-3-3, and a 50 kD protein [46,47,56,59,60]. Conversely, treatment with β_3_-adrenergic receptor agonists increases the phosphorylation of PDE3B associated with caveolin-1 in caveolae, promoting interactions with β_3_-adrenergic receptors, PKA-RII (PKA regulatory subunit), and hormone-sensitive lipase [46,61]. Whether these associations also occur in cardiac myocytes is not yet clear. PDE3B has been reported to weakly associate in a complex containing the unconventional AKAP PI3Kγ, especially with overexpression in HEK293 cells [53,62]. Nonetheless, the significant effects on contractility and rhythm detected in mice lacking PI3Kγ indicate that the determinant of the cardiac phenotype is the ability of PI3Kγ to form more stable complexes with other phosphodiesterases, including PDE3A and PDE4A/B [39].

Of note, however, is that PDE3B can be phosphorylated not only by PKA but also by PKB, the main effector of the PI3K pathway. Although the weak interaction between PI3Kγ and PDE3B reported in the heart awaits further experimental confirmation, severe reduction of the G protein-coupled receptor-driven PI3K pathway by the concomitant loss of PI3Kγ and PI3Kβ has been shown to lead to reduced PDE3B phosphorylation and activity in neurons [63]. Nonetheless, whereas the specific association of PI3Kγ with β-adrenergic signalling in T tubules supports at least a spatial co-localisation of both PI3K and PDE3B [39,53], experiments in knockout mice indicate that PI3Kγ and PDE3B might be part of different functional complexes. On the other hand, the association of PI3Kγ with PKA triggers a negative feedback loop where PKA phosphorylates and inhibits PI3Kγ itself. Interestingly, loss of PDE3B in the heart leads to an unexpectedly strong elevation of cAMP [26], which likely remains spatially confined due to the normal activity of the other phosphodiesterases. Whether this elevated cAMP and PKA activity influences PI3Kγ catalytic activity and, indirectly, the PKB signalling axis is yet to be determined. As enhanced PI3Kγ signalling is involved in the downregulation of β-adrenergic receptor density on the cell surface and, generally, in cardiac myocyte decompensation under stress, PDE3B inhibition could reduce a detrimental signal [64]. Inhibitors specifically distinguishing PDE3B from PDE3A are not currently available, but specific targeting of PDE3B may be therapeutically useful for ischaemia/reperfusion injury. 

## 4. Inotropic Actions of PDE3A Inhibition

As discussed above, PDE3A is part of a multiprotein complex in the sarcoplasmic reticulum through which phospholamban phosphorylation and SERCA2 activity are regulated, and PDE3 inhibition potentiates the stimulatory effects of cAMP on SERCA2 activity in microsomes from cardiac muscle. These effects would be expected to increase contractility by increasing the amplitude of intracellular Ca^2+^ transients. Experiments in mice with selective ablation of PDE3A and PDE3B indicate that inotropic responses to PDE3 inhibition are attributable specifically to inhibition of PDE3A. Phospholamban phosphorylation, SERCA2 activity, intracellular Ca^2+^ cycling, and contractility are increased—and inotropic responses to PDE3 inhibition are eliminated—in *Pde3a*^−/−^ mice; none of these effects are observed in *Pde3b*^−/−^ mice [37]. Furthermore, myocardial contractility in mice is reduced when PDE3A1 is overexpressed [65]. Total intracellular cAMP levels are higher in *Pde3b*^−/−^ mice than in *Pde3a*^−/−^ mice [26], indicating that this is not simply a ‘mass effect’ reflecting the higher abundance of PDE3A relative to PDE3B in cardiac myocytes but is instead due to the specific functional consequences of the intracellular localisation of these isoforms. (A unique role of PDE3B in mitigating reperfusion injury, discussed below, is further evidence along these lines [26].) 

## 5. Pro-Apoptotic Actions of PDE3A Inhibition

PDE3 inhibition in rats and *Pde3a* ablation in mice lead to increases in the phosphorylation of cAMP response element-binding protein (CREB) and consequent increases in the expression of inducible cAMP early repressors (ICER’s), promoting apoptosis [66,67]. Inhibition of PDE3, and especially of PDE3A, by milrinone might involve a phospho-CREB-induced increase in the expression of ICER, with consequent apoptosis and myocardial pathological remodelling. Conversely, there is evidence that increased PDE3A activity, achieved by overexpression in mice, reduces ICER expression, increases Bcl-2 expression, and protects cardiac myocytes against apoptosis [65]. In fact, specific overexpression of myocardial PDE3A1 in transgenic mice confers protection during ischaemia/reperfusion by decreasing cAMP signalling and phosphorylation of CREB, resulting in decreased expression of ICER and reduced apoptosis [65]. PDE3 activity is associated with nuclear membranes in cardiac myocytes [27], and it is possible that the activity localised to this region is responsible for these pro-apoptotic changes in gene expression.

## 6. Pro-Hypertrophic Actions of PDE3A Inhibition

PDE3 inhibition has pro-hypertrophic actions in neonatal rat ventricular myocytes [68]. These effects are essentially reproduced by expression of a dominant-negative form of PDE3A2 induced by a single amino acid substitution in the C-terminal region that renders the protein catalytically inactive but otherwise intact [68]. This dominant-negative construct presumably functions as a competitive inhibitor of the localising protein–protein interactions of the native protein to disrupt its intracellular targeting; this is further evidence that the intracellular targeting of PDE3 isoforms is as important as their catalytic activity. The antihypertrophic effect is especially interesting in view of the description of a set of genetically unrelated missense mutations within a five-amino-acid sequence in PDE3A that increase catalytic activity and lead to a syndrome of brachydactyly and hypertension [69,70]. The hypertension is likely attributable to increased cGMP hydrolysis in vascular smooth muscle; the resulting decrease in intracellular cGMP content would lead to a combination of vasoconstriction and vessel wall hyperplasia. Despite the severe hypertension, however, patients with this syndrome have strikingly low levels of cardiac hypertrophy [71]. This suggests that an increase in PDE3A activity in cardiac myocytes may in fact be antihypertrophic, consistent with the benefits of PDE3A1 overexpression with respect to pathologic remodelling in animal models discussed above [65]. 

## 7. Clinical Experience with PDE3 Inhibition in the Treatment of Heart Disease

With respect to cardiac disease, PDE3 has been of interest principally as a target for increasing contractility in patients with heart failure, a condition in which decreases in β-adrenergic receptor density and increases in Gαi and β-adrenergic receptor kinase activity in cardiac myocytes attenuate cAMP generation lead to decreases in cAMP content, protein phosphorylation and the amplitude of intracellular Ca^2+^ transients [72,73,74,75,76,77,78,79,80,81]. Inhibiting PDE3 has the effect of compensating to some extent for these changes by blocking cAMP hydrolysis and potentiating cAMP-mediated signalling, leading to an increase in myocardial contractility [82,83,84,85,86,87,88].

This short-term benefit, unfortunately, is outweighed by an increase in mortality from sudden cardiac death of ~3% per year when these drugs are administered chronically [4,5,6,7,8,9,10]. The explanation for this increase is unclear (though it seems restricted to patients in whom PDE3 inhibition is used to treat contractile failure; no increase in mortality has been seen when the PDE3 inhibitor cilostazol has been used to treat intermittent claudication [89]). Overexpression of SERCA2 in animal models of ischaemia/reperfusion and chronic heart failure is anti-arrhythmic [90,91]. On the other hand, an increase in the phosphorylation of *L*-type and ryanodine-sensitive Ca^2+^ channels may be pro-arrhythmic [40,41,42,43]. It seems more probable, however, based on the fact that short-term administration of these agents is well tolerated, that mechanisms other than direct pro-arrhythmic actions are involved, and that the pro-apoptotic and pro-hypertrophic effects of PDE3 inhibition described above induce pathologic changes in the myocardium that increase the proclivity toward malignant arrhythmias.

## 8. Selective Targeting of PDE3 Isoforms

The fact that PDE3A and PDE3B have different roles in cardiac myocytes, with PDE3A controlling the pathways responsible for inotropic effects, raises the possibility that targeting PDE3A selectively—or, perhaps even better, selectively targeting one of its three known variants—might improve contractility without increasing sudden cardiac death (as noted above, PDE3A1 is restricted in its distribution to intracellular membranes, so that its inhibition may be less likely to elicit pro-apoptotic and pro-arrhythmic actions [16]). The catalytic activities, substrate affinities, and inhibitor sensitivities of PDE3A1, PDE3A2, and PDE3A3 are identical, making it impossible to selectively target the active sites of individual PDE3A variants [22]. The similarity of the PDE3B active site to the PDE3A active site suggested that selectivity between these two proteins would also prove challenging. Recently, however, investigators described a compound with a tenfold higher affinity for PDE3A relative to PDE3B [92], so this supposition needs to be reconsidered. It is not obvious how targeting all PDE3A isoforms without targeting PDE3B would confer a therapeutic advantage, however. To our knowledge, no compound with a significantly higher affinity for PDE3B relative to PDE3A has been reported.

**Figure 4:**
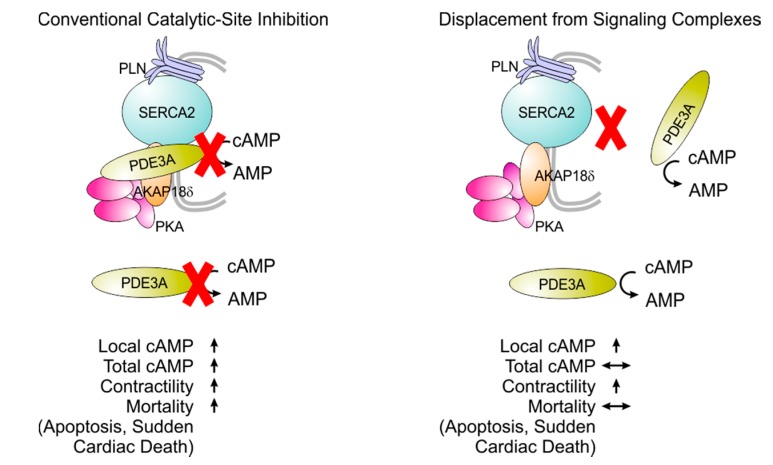
Targeting PDE3A through protein–protein interactions. Conventional PDE3 inhibition activates cAMP-mediated signalling in multiple intracellular compartments, leading possibly to a combination of beneficial and adverse effects. Blocking the protein–protein interactions through which PDE3A is integrated into the SERCA2 complex may stimulate intracellular Ca^2+^ cycling without the adverse effects.

Another possibility is that of targeting individual isoforms not through their active sites but through the protein–protein interactions by which they are localised intracellularly. As noted above, PDE3A1 and PDE3A2 are recruited, by phosphorylation within their N-terminal sequences, to a SERCA2/phospholamban/AKAP/PKA complex in the sarcoplasmic reticulum [37,93]. As a result of this recruitment, PDE3 inhibition has a particularly pronounced effect in potentiating phospholamban phosphorylation and increasing SERCA2 activity, leading to an increase in SERCA2 activity and intracellular Ca^2+^ [38]. Experiments using recombinant proteins have demonstrated direct interactions of PDE3A1 and PDE3A2 with both SERCA2 and AKAP18, and have shown that the interaction of PDE3A1 with SERCA2 is dependent upon its phosphorylation at serine 293, a site within its unique N-terminal extension (Figure 1) [38]. These findings indicate that the protein–protein interactions of the PDE3A isoforms responsible for inotropic responses are highly individualised, opening an avenue to isoform-selective targeting with a high degree of precision. In fact, this approach to isoform-selective targeting—displacing PDE3A from specific multiprotein complexes—has the added benefit of compartment selectivity, which is likely its most important advantage. Conventional PDE3 inhibitors target the enzyme regardless of its intracellular location; this can raise cAMP content globally within the cell, resulting in a combination of pro-apoptotic, pro-hypertrophic, and inotropic [66,67,68]. Blocking the protein–protein interactions that integrate PDE3A into SERCA2 complexes could lead to a selective increase in cAMP content in the vicinity of phospholamban and SERCA2 so as to amplify intracellular Ca^2+^ cycling and increase contractility without the deleterious pro-arrhythmic, pro-apoptotic, and pro-hypertrophic consequences (Figure 4). 

On the other hand, the protein–protein interactions that localise PDE3B intracellularly may be targets through which the cardioprotective effects of PDE3B ablation can be elicited without the adverse pro-hypertrophic and pro-arrhythmic consequences of PDE3A inhibition. The feasibility of this approach has been demonstrated in experiments using peptides that block the protein–protein interactions of cyclic nucleotide phosphodiesterases. In one example, peptide-array scanning identified a cell-permeant peptide based on a sequence in the N-terminus of PDE3B through which it is incorporated into an Epac1/PI3Kγ complex in vascular endothelial cells. This peptide has been shown to block the integration of PDE3B into this complex, increasing the activation of Epac1 by cAMP and promoting intracellular tubule formation, cell adhesion, and cell spreading [94]. 

## 9. Conclusions

Several isoforms in the PDE3 family are expressed in cardiac myocytes, where they have important roles regulating signalling pathways involved in inotropic, pro-apoptotic, and pro-hypertrophic responses. Inhibition of myocardial PDE3 activity is an established therapeutic strategy for increasing contractility in patients with heart failure, but an increase in sudden cardiac death in patients treated chronically with existing PDE3 inhibitors has limited the benefits. Targeting individual PDE3A isoforms in specific intracellular targets could potentially yield inotropic responses without this adverse effect, while targeting PDE3B could have beneficial actions in ischaemia/reperfusion injury. Recent discoveries open the possibility of selectively inhibiting PDE3A or PDE3B at their catalytic sites or, probably more interestingly, of targeting their unique protein–protein interactions to yield compartment-specific effects on intracellular signalling. Time will tell. 

## Abbreviations

aa	amino acid
AKAP	A-kinase-anchoring protein
β_2_-AR	β_2_-adrenergic receptor
cAMP	Cyclic adenosine monophosphate
IRS	Insulin-receptor substrate
NHR	N-terminal hydrophobic region
PLN	Phospholamban
PKA	cAMP-dependent protein kinase
PKA-RII	cAMP-dependent protein kinase regulatory subunit II
PKB (AKT)	Protein kinase B
PKC	Protein kinase C
PI3Kγ	Phosphoinositide 3-kinase γ
PIP3	Phosphatidylinositol (3,4,5)-triphosphate
p110γ	Phosphoinositide 3-kinase γ (PI3Kγ) catalytic subunit
p87	PI3Kγ regulatory subunit (also known as p87PIKAP p87-PI3K adapter protein)
SERCA2	Sarcoplasmic/endoplasmic reticulum calcium ATPase-2
